# The association between cannabis and codeine use: a nationally representative cross-sectional study in Canada

**DOI:** 10.1186/s42238-022-00160-x

**Published:** 2022-09-09

**Authors:** Ria Garg, Kam Shojania, Mary A. De Vera

**Affiliations:** 1 Faculty of Pharmaceutical Sciences, University of British Columbia, 2405 Wesbrook Mall, Vancouver, BC V6T 1Z3 Canada; 2 Collaboration for Outcomes Research and Evaluation, Vancouver, BC Canada; 3 Arthritis Research Canada, Richmond, BC Canada; 4 Division of Rheumatology, Department of Medicine, Faculty of Medicine, University of British Columbia, Vancouver, BC Canada; 5 Centre for Health Evaluation & Outcome Sciences, Vancouver, BC Canada

**Keywords:** Medical cannabis, Codeine, Pain

## Abstract

**Background:**

Due to the growing use of cannabis for the purposes of pain relief, evidence is needed on the impact of cannabis use on concurrent analgesic use. Therefore, our objective was to evaluate the association between the use of cannabis and codeine.

**Methods:**

We conducted a cross-sectional study using data from the nationally representative Canadian Tobacco, Alcohol and Drugs Survey (2017). The primary explanatory variable was self-reported use of cannabis within the past year. The outcome was the use of codeine-containing product(s) within the past year. We used multivariable binomial logistic regression models.

**Results:**

Our study sample comprised 15,459 respondents including 3338 individuals who reported cannabis use within the past year of whom 955 (36.2%) used it for medical purposes. Among individuals who reported cannabis use, the majority were male (*N* = 1833, 62.2%). Self-reported use of cannabis was associated with codeine use (adjusted odds ratio [aOR] 1.89, 95% *CI* 1.36 to 2.62). Additionally, when limited to cannabis users only, we found people who used cannabis for medical purposes to be three times more likely to also report codeine use (adjusted odds ratio [aOR] 2.96, 95% *CI* 1.72 to 5.09).

**Discussion:**

The use of cannabis was associated with increased odds of codeine use, especially among individuals who used it for medical purposes. Our findings suggest a potential role for healthcare providers to be aware of or monitor patients’ use of cannabis, as the long-term adverse events associated with concurrent cannabis and opioid use remain unknown.

## Introduction

Approximately one out of four Canadians aged 15 years and older live with non-cancer chronic pain, a condition for which people may use opioid therapy for adequate pain relief (Reid et al. 2002). However, chronic use of opioids warrants caution due to the increased risk of severe adverse events such as constipation, hyperalgesia, and the potential for developing an opioid addiction, which may result in the occurrence of an overdose (Deshpande et al. 2015; Campbell et al. 2018; Lee et al. 2011). Recent research has shown that concomitant use of cannabis and opioids may be beneficial for chronic non-cancer pain patients, as cannabis may enhance the analgesic effects of opioids, thereby decreasing daily opioid intake and risk of opioid-related harms (Degenhardt et al. 2015; Haroutounian et al. 2016). As a result, cannabis is currently being investigated for the treatment of chronic pain and opioid dependence. While the use of cannabis may offer potential harm reduction to stem the impacts of the current epidemic of opioid-related deaths, little research has been done to assess the long-term patient outcomes associated with concurrent opioid and cannabis use (Rogers et al. 2019; Khan et al. 2020). Specifically, as both substances are central nervous system depressants, concomitant use may put people at increased risk of falls, motor vehicle accidents, and respiratory depression (Rogers et al. 2019; Windle et al. 2021).

Codeine is the most frequently prescribed opioid in the world and is easily accessible to Canadians over the counter (OTC) as a low dose (≤ 15 mg/dosage unit) preparation made in combination with simple analgesics (acetaminophen or nonsteroidal anti-inflammatory drugs [NSAIDs]) upon pharmacist consultation (Nielsen et al. 2018; Non-prescription analgesic and antitussive medications containing codeine: a review of clinical effectiveness and safety 2018; Canadian Institute for Health Information 2019). However, appropriate pharmacy screening for codeine dependence prior to dispensing of low-dose codeine rarely occurs (MacKinnon 2016; Foley et al. 2016). While codeine is primarily indicated for use as a cough suppressant or analgesic, guidelines generally suggest that codeine has a limited role in the management of chronic pain conditions, prior to initiating stronger opioids in acute pain conditions (Gisev et al. 2016). While codeine may be characterized as a “weak opioid,” the risk of dependence and harm secondary to tolerance and subsequent dose escalations must be cautioned (Canadian Institute for Health Information 2019). In fact, the Canadian province of Ontario reported codeine to be detected in 6.3% of all accidental opioid-related deaths that occurred between July 2017 and June 2018 (Chief OAfHPaPPHOOot, Coroner; Ontario Forensic Pathology Service; Ontario Drug Policy Research Network n.d.). Additionally, overuse of codeine-containing products may also lead to serious harms secondary to the supratherapeutic intake of the simple analgesic component. For example, hepatotoxicity may occur due to acetaminophen overdose, and gastric and/or renal complication may arise due to chronic NSAID exposure (Frei et al. 2010).

Due to the legalization of cannabis across Canada (in 2017) and widespread availability of both prescription and OTC codeine, people may be concurrently using cannabis- and codeine-containing products, placing them at increased risk of adverse events (Rogers et al. 2019; Canadian Institute for Health Information 2019; Government of Canada 2021; Corroon et al. 2019). Moreover, healthcare providers may be unaware of their patient’s concomitant cannabis and prescription and/or OTC codeine use as a majority of individuals choose not to disclose their use of cannabis to their healthcare provider due to self-perceived stigma (Leos-Toro et al. 2018). Therefore, the objective of our study was to evaluate the association between the use of cannabis- and codeine-containing products.

## Methods

### Study design and source population

We conducted a population-based cross-sectional study using the public use microdata file from the nationally administered Canadian Tobacco, Alcohol and Drugs Survey (CTADS) as the source population (Statistics Canada 2021). In brief, the CTADS was conducted in 2017 by computer-assisted telephone technology and aimed to assess the prevalence of cigarette smoking, alcohol use, and drug use and the extent of harm related to usage. Survey sampling was allocated based on age, sex, and geographical distribution to ensure representation at both provincial and national levels. Individual participants were 15 years and older and resided in one of the 10 Canadian provinces. Exclusion criteria included residents of the Yukon, Northwest Territories, Nunavut, and individuals living in long-term care institutions or Canadian Forces bases. Additional details on the design and methodology of the CTADS are available through the Statistics Canada (Statistics Canada 2021). Data from the CTADS were kept private and confidential as per the Statistics Act. Where possible, this investigation adhered to STROBE reporting guidelines for observational studies.

### Study variables

The main explanatory variables were as follows: (1) self-reported use of cannabis within the past year (“During the past 12 months have you used marijuana?”) and (2) self-reported use of cannabis for medical purposes within the past year (“In the past 12 months, have you used or tried marijuana (hashish, hash oil or other cannabis derivatives) for medical purposes?”). Both were binary categorical in nature with response options of “yes” or “no.” We also considered additional variables describing cannabis use including the following: (1) method of cannabis consumption (“eaten,” “vaporized,” “drank,” or “smoked”), (2) frequency of cannabis use within the past 3 months (“once or twice,” “monthly,” “weekly,” or “daily or almost daily”), (3) illicit drug use excluding cannabis in the past 12 months (“yes” or “no”), and (4) self-perceived impairment due to cannabis use (“yes” or “no”).

The main outcome variable was self-reported use of any codeine-containing products within the past year. This was assessed by the question “During the past 12 months, have you used any codeine products like Tylenol #3, Tylenol #1, 292’s or 222’s?” with response options of “yes” or “no.”

We considered the following variables as potential confounders to the relationship between cannabis and codeine use: age, sex, geographic region of residence (rural or urban), education (university degree and above, trade/college, secondary, or less than secondary), absence from work (present or absent), current smoking status (smoker or nonsmoker), and self-perceived general health (excellent/very good, good, fair/poor). We selected the aforementioned covariates based on data availability in the CTADS, function as social determinants of health (Canada Go 2020) or due to an established association with cannabis and/or codeine use (Hindocha et al. 2015). Participants with invalid responses (i.e., “don’t know,” “not stated,” or “refusal”) to explanatory, outcome, and confounding variables were excluded.

### Statistical analysis

Descriptive statistics were used to summarize participants’ characteristics. We estimated the weighted prevalence of cannabis use among the Canadian population based on our study sample and described characteristics of cannabis use. In our primary analysis, we used multivariable binomial logistic regression to evaluate the association between self-reported use of cannabis and codeine, adjusted for confounding variables. In the secondary analysis, we limited analysis to cannabis users to evaluate the association between self-reported use of cannabis for medical purposes and codeine. For the secondary analysis, we also used multivariable binomial logistic regression, adjusted for confounding variables. The statistical analyses were weighted using CTADS master weights to provide population-level estimates (i.e., proportion (%), odds ratio (Force 2020)). All of the analyses were completed using SAS University Edition (SAS Institute Inc., Cary, NC).

### Ethics approval

Ethical approval to use the publicly available CTADS data was covered by the University of British Columbia’s Policy (no. LR9) on research involving human participants.

## Results

The CTADS source population consisted of 16,349 participants in total; after excluding 890 participants with invalid responses to study variables, the final study sample included 15,459 individuals. Demographic characteristics for the study sample are reported in Table 1. Overall, 3338 (21.6%) individuals reported use of cannabis within the past year, and 12,121 (78.4%) did not. Among individuals who reported cannabis use, approximately two-thirds were male (*N* = 1833, 62.2%) and between the ages of 15 to 44 years old (*N* = 2967, 74.3%). Illicit drug use was greater among individuals self-reporting use of cannabis (22.3%) in comparison with those who did not (0.8%). Lastly, codeine use was higher among individuals who reported use of cannabis (14.0%) in comparison with those who did not report use of cannabis (8.0%).

**Table 1:** Characteristics of the study sample according to self-reported cannabis use, Canadian Tobacco, Alcohol and Drugs Survey (2017) (*N* = 15,459)

	Cannabis use^**a**^ (***N*** = 3338)	No cannabis use^**b**^ (***N*** = 12,121)
**Sex**		
Male	1833 (62.2)	5554 (46.9)
Female	1505 (37.8)	6567 (53.1)
**Age**		
15–44 years	2967 (74.3)	8560 (43.4)
45+ years	371 (25.7)	3561 (56.6)
**Geographical region**		
Urban	2527 (82.1)	8942 (79.6)
Rural	811 (17.9)	3179 (20.4)
**Education**		
Less than secondary	550 (8.1)	3116 (11.6)
Secondary	1549 (27.8)	4047 (24.9)
Trade/college	811 (34.7)	2826 (29.4)
University and above	427 (29.4)	2132 (34.1)
**Absence from work**^**c**^		
Absent	1095 (30.8)	5291 (42.6)
Present	2243 (69.2)	6830 (57.4)
**Marital status**		
Partner	474 (42.2)	3704 (64.6)
No partner	2864 (57.8)	8417 (35.4)
**Smoking status**		
Nonsmoker	2276 (62.3)	11,133 (89.5)
Smoker	1062 (37.7)	988 (10.5)
**Codeine use**^**d**^		
No	2876 (86.0)	11,130 (92.0)
Yes	462 (14.0)	991 (8.0)
**Alcohol consumption**^**d**^		
Abstainer/low	3034 (88.3)	11,879 (97.2)
Hazardous/harmful	304^f^ (11.7)	242^f^ (2.8)
**Illicit drug use**^**e**^		
No	2624 (77.7)	12,001 (99.2)
Yes	714 (22.3)	120^f^ (0.8)
**Self-perceived health**		
Very good/excellent	2096 (57.8)	8597 (66.1)
Good	983 (33.2)	2883 (25.9)
Fair/poor	259^f^ (9.0)	641 (8.0)

^a^Use of cannabis-containing products in the past 12 months

^b^No use of cannabis-containing products in the past 12 months

^c^During the week prior to the date survey was conducted

^d^Use over the past 12 months

^e^Includes cocaine, speed/meth, ecstasy, hallucinogens, salvia, heroin, inhalants, abuse of pain relievers, stimulants, and sedatives to get high in the past 12 months

^f^Coefficients of variation are within the range of 16.6 to 33.3%. Therefore, it is required to report by the Statistics Canada that estimates are associated with high levels of error [21]. All percentages reported are weighted using probability weights from Statistics Canada

Patterns of cannabis used among individuals who did and did not self-report use of codeine-containing product(s) are stated in Table 2. The vast majority of individuals who did (98.3%) and did not (97.4%) self-report use of codeine also reported using cannabis more than once. However, the self-reported prevalence of cannabis on a weekly or more frequent basis was greater among individuals who reported use of codeine (55.4%), in comparison with those who did not report use of codeine (36.0%). Additionally, among individuals who reported use of codeine, a higher proportion also reported use of cannabis for medical purposes (61.6%), in comparison with those who did not report use of codeine (32.1%).

**Table 2:** Characteristics of cannabis use among codeine users and nonusers, Canadian Tobacco, Alcohol and Drugs Survey (2017), study sample (*N* = 3338)

	Codeine use^**a**^ (***N*** = 462) *N* (%)	No codeine use^**b**^ (***N*** = 2,875) *N* (%)
**Used cannabis more than once**		
No	18^f^ (1.7)	163^e^ (2.6)
Yes	444 (98.3)	2712 (97.4)
**Cannabis use for medical purposes**		
No	271 (38.3)	2112 (67.9)
Yes	191 (61.6)	764 (32.1)
**Method of cannabis use**^**c**^		
Smoked	434 (90.8)	2708 (89.7)
Eaten	233^e^ (51.4)	1121 (36.6)
Vaporized	188^e^ (43.8)	869 (27.5)
Drank	64^f^ (16.3)	239 (7.4)
**Frequency of cannabis use**^**d**^		
Monthly or less	127^e^ (22.8)	922 (35.7)
Weekly or more	207^e^ (55.4)	981 (36.0)
Not stated	120 (21.8)	969 (28.3)
**Self-perceived impairment of ability to complete daily tasks due to cannabis use**		
No	370 (80.1)	2419 (84.1)
Yes	74^f^ (16.0)	289^e^ (10.1)

^a^Use of cannabis-containing products in the past 12 months

^b^No use of cannabis-containing products in the past 12 months

^c^Cumulative percentage may be greater than 100, as multiple categories may be relevant to each participant

^d^Use over the past 3 months

^e^Coefficients of variation are within the range of 16.6 to 33.3%. Therefore, it is required to report by the Statistics Canada that estimates are associated with high levels of error (Statistics Canada 2021)

^f^Coefficients of variation are greater than 33.3%. Therefore, estimates do not meet Statistics Canada’s quality standards. Conclusions based on this data will be unreliable and most likely invalid (Statistics Canada 2021). All percentages reported are weighted using probability weights from Statistics Canada

The unadjusted odds ratio (OR) for the association between self-reported use of cannabis and codeine was 1.86 (95% *CI*, 1.37 to 2.54). After including age, sex, geographic region of residence, education, absence from work, smoking status, marital status, and self-perceived health in the multivariable logistic regression model, we observed a small increase in the OR representing the association between cannabis and codeine use (adjusted odds ratio [aOR] 1.90, 95% *CI* 1.38 to 2.62) (Table 3). In this multivariable model, we also found an inverse association between being in very good/excellent health with use of codeine (*aOR* 0.53, 95% *CI* 0.36 to 0.80) as compared to being in fair/poor health.

**Table 3:** Unadjusted and adjusted logistic regression models evaluating the association between cannabis and codeine use among the Canadian Population, Canadian Tobacco, Alcohol and Drugs Survey (2017)

	Overall (*N* = 15,459)
	Codeine use^**a**^(yes vs no) OR (95% *CI*)
**Unadjusted model**	
**Cannabis**^**b**^	
No	Reference
Yes	**1.86 (1.37, 2.54)**
**Multivariable model**	
**Cannabis**^**b**^	
No	Reference
Yes	**1.90 (1.38, 2.62)**
**Sex**	
Female	Reference
Male	0.83 (0.63, 1.10)
**Age**	
15–44 years	0.79 (0.58, 1.09)
45+ years	Reference
**Geographic region**	
Rural	Reference
Urban	1.02 (0.76, 1.37)
**Education**	
University and above	Reference
Trade/college	1.26 (0.87, 1.82)
Secondary	1.01 (0.71, 1.44)
Less than secondary	0.90 (0.56, 1.44)
**Absence from work**^**c**^	
Absent	Reference
Present	1.10 (0.82, 1.48)
**Marital status**	
No partner	Reference
Partner	0.91 (0.68, 1.22)
**Smoking status**	
Nonsmoker	Reference
Smoker	1.03 (0.73, 1.44)
**Self-perceived general health**	
Fair/poor	Reference
Good	0.72 (0.47, 1.11)
Very good/excellent	**0.53 (0.36, 0.80)**

Abbreviations: *OR* odds ratio, *CI* confidence interval. Estimates are probability weighted using weights from Statistics Canada

^a^Use of any codeine-containing products in the past 12 months

^b^Use of cannabis containing products in the past 12 months

^c^During the week prior to the date survey was conducted

For our secondary analyses limited to individuals who used cannabis, the unadjusted OR for the association between self-reported use of cannabis for medical purposes and codeine-containing product(s) was 3.42 (95% *CI*, 2.04 to 5.76). In the multivariable model, after adjusted for age, sex, geographic region of residence, education, absence from work, smoking status, marital status, and self-perceived health, we observed an attenuation of the OR but nonetheless represented a nearly 3-fold increase in the odds of codeine use among individuals who used cannabis for medical purposes (*aOR* 2.96, 95% *CI* 1.71 to 5.10) (Table 4). Additionally, within this subgroup of cannabis users, males had lower odds of reporting codeine use in comparison with females (*aOR* 0.57, 95% *CI* 0.34 to 0.97).

**Table 4:** Unadjusted and adjusted logistic regression models evaluating the association between cannabis use for medical purposes and codeine use among the Canadian Population, Canadian Tobacco, Alcohol and Drugs Survey (2017)

	Cannabis users (*N*=3338)
	Codeine use^**a**^ (Yes vs No) OR (95% CI)
**Unadjusted model**	
**Cannabis for medical purposes**^**b**^	
No	Reference
Yes	**3.42 (2.04, 5.76)**
**Multivariable model**	
** Cannabis for medical purposes**^**b**^	
No	Reference
Yes	**2.96 (1.71, 5.10)**
**Sex**	
Female	Reference
Male	**0.56 (0.34, 0.97)**
**Age**	
15–44 years	0.71 (0.39, 1.29)
45+ years	Reference
**Geographic region**	
Rural	Reference
Urban	0.73 (0.41, 1.33)
**Education**	
University and above	Reference
Trade/college	1.89 (0.85, 4.19)
Secondary	1.75 (0.82, 3.75)
Less than secondary	1.21 (0.48, 3.05)
**Absence from work**^**c**^	
Absent	Reference
Present	1.05 (0.60, 1.86)
**Marital status**	
No partner	Reference
Partner	0.58 (0.32, 1.03)
**Smoking status**	
Nonsmoker	Reference
Smoker	1.22 (0.71, 2.11)
**Self-perceived general health**	
Fair/poor	Reference
Good	1.32 (0.63, 2.78)
Very good/excellent	1.08 (0.51, 2.31)

Estimates are probability weighted using weights from Statistics Canada

Abbreviations: *OR* odds ratio, *CI* confidence interval

^a^Use of any codeine-containing products in the past 12 months

^b^Use of cannabis-containing products in the past 12 months for medical purposes

^c^During the week prior to the date survey was conducted

## Discussion

We used data from an established nationally representative Canadian survey to evaluate the relationship between cannabis and codeine use. Overall, 21.6% of Canadians self-reported use of cannabis in 2017, of whom 36.2% used cannabis for medical purposes. Multivariable logistic regression models indicate that individuals who self-reported use of cannabis had greater odds of also reporting use of codeine. When limited to cannabis users, individuals who used cannabis for medical purposes were nearly three times more likely to also report codeine use. These findings reflect the increasing need to further investigate the safety profile of concomitant cannabis and opioid use along with the need for healthcare providers, particularly prescribers (i.e., physicians, nurse practitioners) and pharmacists to assess a patient’s use of cannabis prior to prescribing or dispensing codeine. Furthermore, these findings suggest a potential role for healthcare providers for monitoring patients’ use of cannabis, as the long-term adverse events associated with concurrent use with prescription medications, particularly codeine, remain unknown.

Evidence to support the use of cannabis for treatment of non-cancer chronic pain and opioid dependence has recently emerged, as studies highlight a declining trend in opioid consumption following subsequent cannabis use (Lucas et al. 2021; Dranitsaris et al. 2021). Specifically, a Canadian prospective study (*N* = 1145) conducted by Lucus et al. reported an overall 78% reduction in mean opioid dosage over the course of 6 months with cannabis use (Lucas et al. 2021). However, it should be noted that study participants received physician guidance regarding appropriate cannabis use throughout the entirety of the study, an aspect which is not observed in the general population (Leos-Toro et al. 2018; Lucas et al. 2021). Additionally, due to the short-term durations (e.g., 6 months) of studies assessing the impact of cannabis on opioid consumption, many fail to assess the potential for long-term adverse effects. Furthermore, with concomitant use, it is important to consider harms associated with polypharmacy, which has always been shown to be more significant than single drug use (Lake et al. 2020; Giummarra et al. 2015).

Our study adds to the existing body of literature by highlighting the strong positive association between the use of cannabis and codeine, two substances which are widely accessible to patients without prescription and/or healthcare professional supervision, particularly in recent years in Canada (Novak et al. 2016). To contextualize with studies in other jurisdictions, in 2018, Caputi et al. used nationally representative individual-level data and reported positive association (*aOR* 1.66 95% *CI* 1.49 to 1.83) between use of cannabis and pain relieving medications (Caputi and Humphreys 2018), similar to our current study. Additionally, our study results reflect a strong association (3-fold odds) between the use of cannabis for medical purposes and codeine use. While our study was unable to specify which medical condition study participants were using medical cannabis for, this substantial increase in odds of codeine use among individuals who used cannabis for medical purposes may be attributed to experiencing greater pain severity as a majority of people who use cannabis for medical purposes do so for pain relief (Lake et al. 2019; Boehnke et al. 2022; Leung et al. 2022).

In addition to findings on the association between cannabis and opioid use, our findings also highlight that people who report very good/excellent perceived general health were less likely to report codeine use, compared to those who perceive their health as fair/poor. These findings are in line with previous literature demonstrating poorer physical and mental health scores among individuals who report opioid use (Millson et al. 2004). Another unique finding of our study was that among cannabis users, males were less likely to use codeine in comparison with females. These findings are in line with previous literature that highlights differences in opioid prescription rates among males and females. In comparison to males, females are more likely to be prescribed opioids (Serdarevic et al. 2017), which may be explained in part due to the increased prevalence of chronic conditions resulting in pain among females, such as inflammatory arthritis, fibromyalgia, and migraines (Darnall et al. 2012).

Limitations must be considered when interpreting the results of this study. First, the CTADS survey was conducted between February and December of 2017; therefore, the survey was conducted prior to legalization of cannabis across Canada (October 17, 2018) which may put study results at risk of respondent or social desirability bias. However, it should be noted that access to cannabis for medical purpose has been legalized in Canada since 2000. While we do acknowledge the rising prevalence of cannabis use since legalization in 2018 (Rotermann 2021), the intent of our work was not to establish cannabis use patterns but rather to explore the potential for concurrent cannabis and codeine use. Future research is required to further explore the relationship and impacts of concurrent cannabis and opioid use. Second, our study is restricted to the variables included in the CTADS survey, which does not include information on how frequently participants consumed codeine-containing product(s), limiting our results from truly assessing concurrent use. Third, the cross-sectional study design precludes the ability to infer temporality between exposure and outcome variables. Fourth, exclusion of survey participants with invalid responses to explanatory, outcome, and confounding variables may have impacted the external validity of our study, as a greater proportion of participants included in our study received education past secondary school in comparison with those excluded, which may bias the result of our analysis. Fourth, we excluded survey participants (*n* = 890) with invalid responses to explanatory, outcome, and confounding variables. Comparison with our study sample on sociodemographic characteristics (i.e., sex, age, geographic region, and education) revealed no differences except with respect to education, whereby a lower proportion of excluded participants reported completing secondary school education or did not provide a response to the question. Lastly, due to sample size limitations, certain descriptive variables did not meet Statistics Canada’s definition for acceptable “quality,” as the coefficient of variation was above their stated threshold (Statistics Canada 2021). Therefore, we were unable to include covariates that have been shown to be associated with codeine use such as use of psychoactive substances, cannabis use characteristics (i.e., method, frequency, self-perceived impairment, and reason for medical cannabis use), and alcohol consumption to maintain the quality of our analysis.

## Conclusion

Altogether, this current study suggests that individuals who have used cannabis in the past year are at 72% greater odds of reporting the use of codeine-containing product(s). When limited to individuals who used cannabis in the past year, those who used cannabis for medical purpose were at an even greater odds of codeine use and were three times more likely to use codeine-containing product(s) in comparison with those who used cannabis for nonmedical purposes. The strong association between codeine and cannabis indicates the need for health professionals to monitor patients for concomitant use of cannabis and codeine as long-term adverse events associated with concomitant use remain largely unknown. Patients using cannabis for medical purposes may benefit from interdisciplinary care and should receive patient counseling on the potential for increased central nervous system depression if cannabis and opioids are used concurrently (Rogers et al. 2019).

## Data Availability

The public use microdata file can be accessed through the Statistics Canada (https://www23.statcan.gc.ca/imdb/p2SV.pl?Function=getSurvey&SDDS=4440).

## Abbreviations

OTC: Over the counter
NSAIDs: Nonsteroidal anti-inflammatory drugs
CTADS: Canadian Tobacco, Alcohol and Drugs Survey
OR: Odds ratio
aOR: Adjusted odds ratio
